# The effects of mentor support on Ed.D students’ research creativity: mediating roles of research self-efficacy and learning engagement

**DOI:** 10.3389/fpsyg.2025.1600533

**Published:** 2025-06-25

**Authors:** Haifei Miao, Lixing Zhao, Zhigang Wang

**Affiliations:** ^1^School of Education, Shaanxi Normal University, Xi’an, China; ^2^Faculty of Education and Liberal Studies, City University of Malaysia, Kuala Lumpur, Malaysia

**Keywords:** mentor support, research self-efficacy, learning engagement, research creativity, doctor of education

## Abstract

**Introduction:**

Doctor of education (Ed.D) is a professional degree designed to cultivate highly specialized professionals in education, teaching, and educational leadership. Ed.D holds a crucial position within the higher education system, with its core mission being the cultivation of high-quality talent who possess strong research creativity. However, limited research has specifically addressed how to enhance the research creativity of Ed.D students in higher education.

**Methods:**

This study utilized a structural equation modeling approach to investigate the influence of mentor support on Ed.D students research creativity, with a focus on the chain mediating effects of research self-efficacy and learning engagement. A sample of 366 Chinese Ed.D students was surveyed using validated scales for mentor support, research self-efficacy, learning engagement, and research creativity.

**Results:**

The findings indicate that mentor support is significantly positively correlated with the research creativity of Ed.D students; research self-efficacy and learning engagement serve as mediators in the relationship between mentor support and research creativity. Moreover, the average scores for Ed.D students’ research self-efficacy and learning engagement are comparatively low, with male students reporting higher levels of research self-efficacy and research creativity on average.

**Discussion:**

These results provide empirical support and practical recommendations for enhancing the research ability of Ed.D students.

## 1 Introduction

Given the increasing importance of Ed.D (Doctor of Education) programs, it is crucial to investigate strategies for enhancing the research abilities of these students, a challenge that higher education institutions must address (Foster et al., 2023; The Carnegie Project on the Education Doctorate, 2024). The Ed.D program is designed to transform experienced practitioners into practitioner-researchers who are capable of developing, implementing, evaluating, and refining programs in their professional environments, grounded in empirical research (Kerrigan and Hayes, 2016; The Carnegie Project on the Education Doctorate, 2024). Since the 1990s, professional doctorates have rapidly proliferated in countries such as the United States, the United Kingdom, Australia, and New Zealand, emerging as a key component of the global graduate education landscape (Bourner et al., 2001; Kot and Hendel, 2012). Increasingly, countries worldwide are recognizing the value of professional doctorates, leading to the introduction and expansion of these degrees (Zambo et al., 2014). In China, with strong policy support, the Ed.D has also experienced rapid growth in both enrollment and the number of institutions authorized to offer the degree.

Ed.D programs were first introduced in China in 2010 (Li et al., 2024). In 2009, China’s National Office for Academic Degrees issued the “Plan for Professional Degree Establishment of Doctorate in Education,” which outlined that the Ed.D was primarily designed for educational practitioners across various roles, including school teachers, principals, teacher educators, student counselors, administrators, and policymakers (Gong et al., 2022). In alignment with China’s ambition to develop world-class universities and foster social development, professional doctoral programs, such as the Ed.D, have been strongly supported and encouraged by the government. Over the past decade, the Ed.D programs in China has undergone rapid development, with a continuously expanding enrollment. As of 2023, a total of 8,467 students have been admitted (Qin et al., 2025). As graduate education rapidly expands in China, the focus has shifted to enhancing the quality of education and fostering research creativity among students. A major challenge facing Ed.D education in China is how to improve the quality of training while maintaining this growth in scale (Qin and Wu, 2024). Chinese graduate students are currently facing challenges in research creativity, including a lack of original research outcomes, insufficient transformation of research results, and low levels of participation in academic activities (Yao and Yu, 2019; Li and Hu, 2024). Therefore, there is an urgent need to enhance their capacity for research creativity for building a high-quality graduate education system. Training more and more high-quality talents has become an important mission of graduate education in the new era (Knutson et al., 2022). Accordingly, balancing the expansion of Ed.D programs with the improvement of training quality presents a significant challenge for China’s higher education system.

Studies have highlighted the growing importance of mentor support in Ed.D programs, but few have specifically examined how mentors influence students’ innovative abilities (Geesa et al., 2020). Several scholars have emphasized the increasing significance of mentorship in graduate and professional education (Snijders et al., 2020; Su et al., 2022). Mentorship plays a critical role during doctoral studies, with multiple mentoring relationships contributing significantly to success in the Ed.D program (Amador-Campos et al., 2023). Teacher support has also been shown to significantly impact academic achievement in university students (Huang and Wang, 2023). However, while recent studies have explored the motivations of Ed.D candidates, the interaction between internal and external factors remains largely unexplored (Gong et al., 2022). How to effectively stimulate postgraduate student creativity has attracted increasing interests of both scholars and educators (Shang et al., 2024). Additionally, there is a gap in research regarding the experiences of Ed.D students, particularly how learning environments affect various dimensions of student engagement (Sökmen, 2021).

This study seeks to fill existing gaps by examining the impact of mentor support on Ed.D students’ research creativity, with a particular focus on the roles of research self-efficacy and learning engagement. In this context, the chain mediation model proposed in this study offers a framework for understanding the roles of research self-efficacy and learning engagement in enhancing Ed.D students’ research creativity through mentor support. The findings offer valuable insights for educators and institutions, guiding the design of instructional strategies and professional development programs aimed at Ed.D students.

## 2 Literature review

### 2.1 Mentor support

Mentor support encompasses the psychological, academic, and resource-related assistance mentors provide to students, including emotional encouragement, academic guidance, and feedback (Lee, 2007). This support can be categorized into three types: academic support, personal support, and autonomy support (Overall et al., 2011). Academic support involves mentors actively assisting students in their scholarly activities, offering timely feedback, and guiding them through research processes. This type of support directly enhances students’ research capabilities. Personal support, on the other hand, includes emotional encouragement and confidence-building, helping students navigate challenges and remain motivated in their academic pursuits. Finally, autonomy support creates an environment where students feel encouraged to share their ideas freely and are given the autonomy to make independent decisions. This fosters an atmosphere that promotes creative thinking and innovation in research.

Graduate training often relies on a mentor responsibility system, where mentor support plays a pivotal role in enhancing students’ academic and research capabilities (Choy et al., 2015). Mentor support provides essential resources, such as timely feedback and guidance, which are critical for fostering research innovation and academic growth (Ren and Li, 2024). For Ed.D students, mentoring relationships are especially vital. They offer the diverse perspectives and emotional support needed to navigate the complexities of doctoral research. Mentors encourage students to independently develop research questions, tackle academic tasks, and refine problem-solving skills, thus significantly contributing to their research competence (Mullen and Klimaitis, 2021). Furthermore, mentor support is instrumental in boosting students’ self-efficacy, which in turn increases their engagement in the learning process and research activities (Ren and Li, 2024). By sharing their academic expertise and personal experiences, mentors also contribute significantly to students’ professional development, helping them advance in their respective research fields. Ultimately, mentor support fosters research creativity, primarily through its positive effect on students’ self-efficacy, which mediates their research innovation.

### 2.2 Research creativity

In the realm of research, creativity is understood as the capacity to generate, develop, implement, and communicate innovative and valuable ideas, products, methods, or processes. Scott and Bruce (1994) conceptualize individual creative behavior as a process that unfolds in three stages: idea generation, promotion, and realization. Wadaani (2015) extends this definition, describing creativity as the ability to synthesize existing knowledge and ideas to create new concepts that drive innovation. Research creativity, therefore, involves not only the generation of novel ideas but also the refinement and application of those ideas in ways that bring practical, functional, and effective solutions to research challenges. Existing research highlights the significant impact that mentor support has on doctoral students’ research creativity and academic outcomes (Gruzdev et al., 2020). Mentor support plays a crucial role by acknowledging students’ ideas and contributions, which boosts their confidence and fosters open communication. This, in turn, encourages students to engage more frequently in discussions with their mentors and actively participate in learning-oriented activities, ultimately helping them achieve specific academic goals.

Research creativity is critical for technological innovation and knowledge production (Zhang Y. et al., 2024; Zhang B. et al., 2024). Understanding the key factors that influence doctoral students’ creativity is essential for identifying challenges within graduate education and fostering the development of student innovation (Gu et al., 2017). While intrinsic motivation drives creativity, it is also nurtured by an environment that offers challenging research opportunities, as well as substantial support and constructive feedback from mentors and peers (Liu Q. et al., 2023). However, despite its importance, there is limited understanding of how mentors specifically influence graduate student creativity within academic organizations (Gu et al., 2017). Most research in doctoral education focuses on the role of the mentor and the supervision process, with relatively few studies examining how creativity itself is cultivated and supported throughout the doctoral journey.

### 2.3 Research self-efficacy

Self-efficacy refers to an individual’s subjective belief in their ability to utilize knowledge, skills, and experience to successfully complete specific tasks (Bandura, 1986a; Bandura, 1986b). In the context of scientific research, research self-efficacy specifically reflects an individual’s confidence in their capacity to engage in various research activities, such as study design and data analysis (Hemmings and Kay, 2016). Rooted in social cognitive theory, research self-efficacy highlights an individual’s belief in their ability to perform academic research tasks. It encompasses the confidence to carry out key aspects of the research process, including conducting literature reviews, collecting and analyzing data, and writing research reports (Van Dinther et al., 2011; Fokkens-Bruinsma et al., 2021). Based on these definitions, this study defines research self-efficacy as Ed.D students’ confidence in their ability to successfully complete research tasks and achieve research goals using the skills and abilities they have acquired.

Previous studies have demonstrated that graduate students’ research self-efficacy is significantly influenced by their academic environment, including mentor guidance and institutional systems. Adequate mentor support is critical in strengthening doctoral students’ research self-efficacy, which, in turn, enhances their research capabilities (Curtin et al., 2016). The mentor-student relationship plays a pivotal role in determining academic success, as it fosters both intellectual and emotional support, which are essential for overcoming the challenges of doctoral research (Russo, 2011; Overall et al., 2011). Furthermore, research has consistently shown that a strong mentoring relationship is linked to increased self-confidence and persistence in the face of research challenges. As students receive constructive feedback and guidance, they are better able to approach research tasks with confidence and resilience (Cobb et al., 2020; Cai et al., 2023). Moreover, research self-efficacy plays a crucial role in the development of research creativity, acting as a key factor in both research creativity and productivity (Kahn and Scott, 1997; Pasupathy and Siwatu, 2014). High levels of research self-efficacy enable students to confidently identify research problems, develop appropriate methodologies, and achieve successful research outcomes (Sökmen, 2021).

### 2.4 Learning engagement

Learning engagement is commonly defined as the degree of cognitive, emotional, and behavioral involvement that students invest in their scientific research activities. Kuh (1991) first introduced the concept of student engagement in higher education, emphasizing its significance in promoting student learning and development. Schaufeli et al. (2002) expanded on this by conceptualizing engagement as a positive psychological state characterized by dedication, absorption, and vigor, all of which are essential for sustained academic involvement. Fredricks et al. (2004) further developed the concept, identifying three key dimensions of learning engagement: behavioral, cognitive, and emotional engagement. Behavioral engagement encompasses active participation in academic activities such as attending lectures, completing assignments, and adhering to class rules. Emotional engagement refers to students’ positive feelings toward teachers, classmates, academic activities, and their sense of belonging to the academic community (Appleton et al., 2006). Cognitive engagement, on the other hand, involves students’ commitment to learning, including self-regulation, persistence, and the effort required to understand complex concepts or master challenging skills (Fredricks et al., 2016). This three-dimensional framework of learning engagement has gained widespread acceptance and has been instrumental in understanding students’ academic experiences. Researchers have further emphasized that learning engagement is not only about active participation in course-related activities but also the time and effort students dedicate to these activities (Bakker et al., 2015; Perkmann et al., 2021).

The existing literature highlights the significant role of mentor support in promoting meaningful engagement in coursework (Pineda-Báez et al., 2019), academic motivation and sustaining students’ academic progress (Patrick et al., 2007). Specifically, students who perceive strong support from their mentors are more likely to demonstrate positive academic beliefs and invest greater effort in their coursework (Liu C. et al., 2023). Mentor support has been shown to function as a crucial social influence in shaping student engagement, where teachers who provide care, encouragement, constructive feedback, and personalized learning support significantly enhance students’ academic involvement and achievement (Sadoughi and Hejazi, 2023; Amerstorfer and Freiin von Münster-Kistner, 2021). Moreover, learning engagement has been identified as a mediating factor that links instructional strategies to improved student performance, reinforcing the idea that active involvement is essential for academic success (Guthrie et al., 2000; Mayer et al., 2009; Heilporn et al., 2021; Chen et al., 2024). Empirical evidence further supports this, demonstrating that learning engagement mediates the relationship between positive emotions and academic performance, emphasizing its role in facilitating favorable educational outcomes (Carmona-Halty et al., 2021). Additionally, student engagement plays a key role in mediating the impact of perceived teacher support on student achievement (Tao et al., 2022).

## 3 The present study

**Figure 1:**
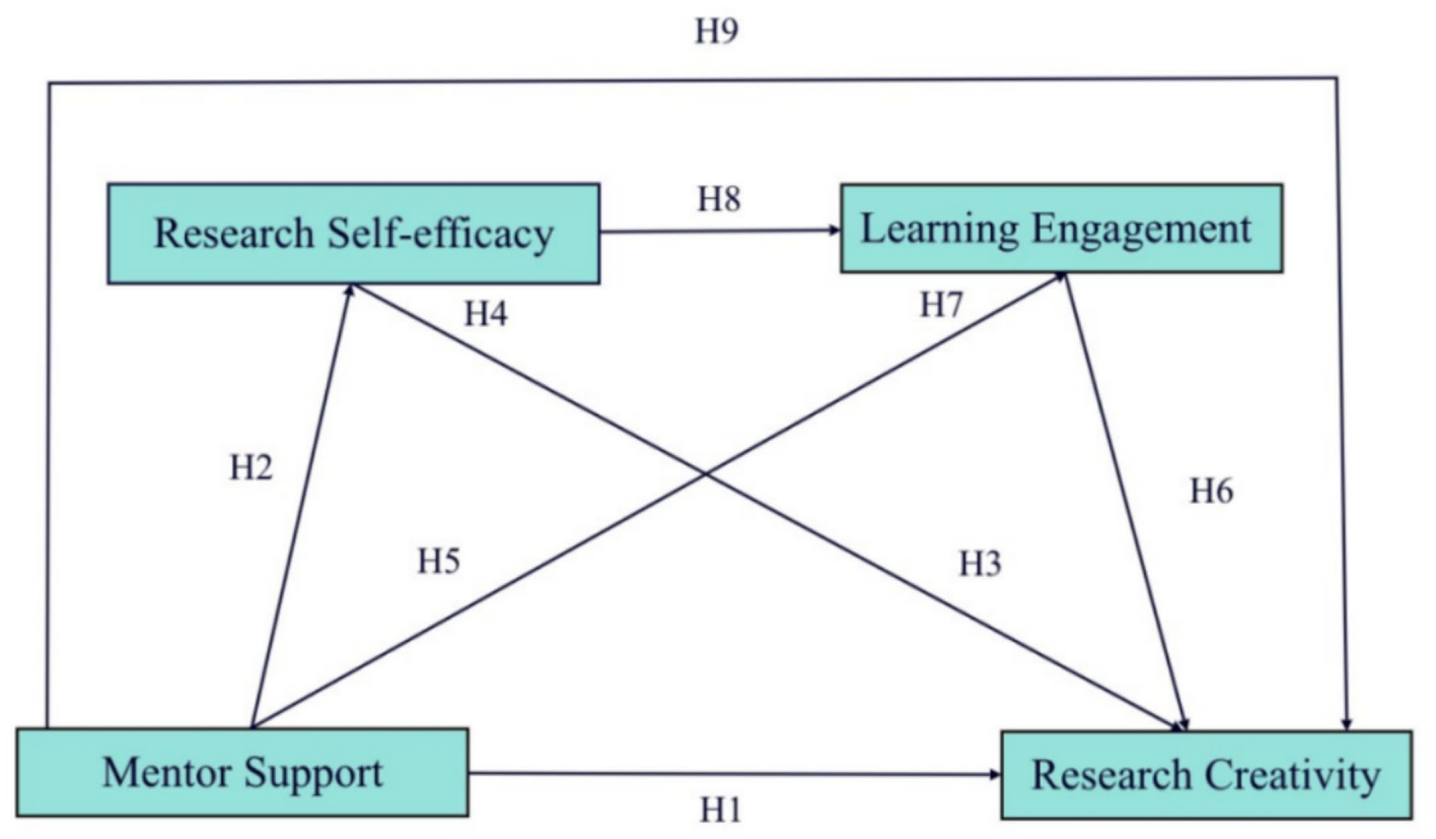
Theoretical model.

Building on the framework of social cognitive theory and extending previous research, this study proposes a model to explore how mentor support influences research creativity among Ed.D students (Figure 1). According to social cognitive theory, human actions are influenced by the interplay of personal, environmental, and behavioral factors (Bandura, 1986a; Bandura, 1986b; Bandura, 2001; Schunk and Pajares, 2002). Triadic reciprocal determinism and self-efficacy are key and core components of social cognitive theory. A positive school climate that fosters interpersonal relationships and a sense of belonging contributes to higher academic self-efficacy and, consequently, better academic outcomes (Zysberg and Schwabsky, 2021). This theory is particularly relevant in the context of Ed.D programs, where students’ research creativity and academic success are significantly influenced by their perceived research competence, learning engagement, and the support they receive from mentors and the institutional environment.

While existing literature has examined the impact of positive factors such as self-efficacy and learning engagement on graduate students’ innovation abilities, limited research has focused on how mentor support enhances these factors to ultimately foster research creativity in Ed.D students. This gap highlights the need to explore how to effectively enhance the research abilities of Ed.D students, an emerging challenge for higher education institutions (Foster et al., 2023). Most studies on research capacity have been conducted in Europe and the United States, with limited research focusing on Chinese samples (Han et al., 2024). This gap in the literature is addressed by examining the mediating roles of research self-efficacy and learning engagement in the relationship between mentor support and research creativity. The study aims to investigate the influence of mentor support on Ed.D students’ research creativity, with a focus on the chain mediating effects of research self-efficacy and learning engagement. By understanding these relationships, the research seeks to offer insights for educational institutions on how to refine graduate training programs, specifically focusing on promoting innovation and creativity among Ed. D students.

Based on this structure, nine hypotheses were subsequently developed.

*H1*: Mentor support is positively associated with Ed.D research creativity

*H2*: Mentor support is positively associated with Ed.D research self-efficacy.

*H3*: Research self-efficacy has a positive relationship with Ed.D research creativity.

*H4*: Research self-efficacy has a mediating effect between mentor support and Ed.D research creativity.

*H5*: Mentor support is positively associated with Ed.D learning engagement.

*H6*: Learning engagement has a positive relationship with Ed.D research creativity.

*H7*: Ed.D learning engagement has a mediating effect between mentor support and research creativity.

*H8*: Ed.D Research self-efficacy has a positive relationship with learning engagement

*H9*: Ed.D research self-efficacy and learning engagement has chain mediating effects between mentor support and research creativity.

## 4 Methods

### 4.1 Participants

Participants in this study were recruited through the questionnaire star platform, a widely used data collection tool in China. The target population comprised Ed.D students from two universities directly administered by the Ministry of Education of the People’s Republic of China. University A and University B are two leading normal universities in China and are part of the “Double First-Class” initiative, which aims to develop world-class universities and disciplines. Their academic prestige and strong capacity for doctoral education make them highly representative of Ed.D programs in China. Moreover, these two normal universities enroll the largest number of Ed.D students nationwide. This substantial enrollment provides a robust and reliable sample for empirical analysis. In addition, the geographical distribution of these institutions ensures regional diversity. University A is located in the eastern region of China, while University B is in the western region. This selection helps capture the influence of regional educational contexts and accounts for geographic variability in student experiences and institutional environments.

Stratified random sampling was employed to ensure a representative sample from various regions and academic disciplines. The total population of Ed.D students was stratified based on two key variables: grade and major. These stratification criteria were chosen because they may influence students’ perceptions of mentor support, research self-efficacy, and learning engagement. Based on the stratification criteria, the full population of Ed.D students was grouped into homogeneous subgroups to form the sampling frame. Within each stratum, participants were selected using a random number generator to ensure that the sampling process was unbiased. A total of approximately 392 questionnaires were distributed, and 366 valid responses were collected. The final sample maintained a balanced representation across different years and areas of specialization, ensuring the generalizability of the findings. This approach ensured that all subgroups of the Ed.D population were proportionally represented. The survey link was distributed to over 20 Ed.D student groups on WeChat, with the cooperation of university faculty and student mentors. Prior to participation, all respondents were provided with informed consent forms and detailed information about the study. Data collection was conducted via the questionnaire star platform, and ethical approval for the study was obtained prior to data collection.

This study was conducted in accordance with the ethical standards of the Declaration of Helsinki. All participants were informed about the purpose of the study, the voluntary nature of their participation, and their right to withdraw at any time without any consequences. Informed consent was obtained before participants began the questionnaire. To maintain anonymity, no personal identifying information was collected, and all responses were treated with strict confidentiality. As shown in Table 1, 190 samples were collected from A Normal University, representing 51.91%, while 176 samples were collected from B Normal University, accounting for 48.09%. The gender distribution was relatively balanced, with males comprising 46.45% and females 53.55%. The majority of participants were between 36 and 40 years old, making up 39.10% of the sample. In terms of grade level, first-year students accounted for 24.32%, second-year students 25.68%, third-year students 25.14%, and fourth-year students 24.86%, indicating a fairly even distribution across academic years.

**Table 1:** Sample distribution.

Variables	Items	Frequency	Percentage
University	A Normal University	190	51.91%
	B Normal University	176	48.09%
Gender	Male	170	46.45%
	Female	196	53.55%
Age	less than 30	33	9.00%
	30–35	98	26.80%
	36–40	143	39.10%
	Greater than 41	92	25.10%
Grades	First year	89	24.32%
	Second year	94	25.68%
	Third year	92	25.14%
	Fourth year	91	24.86%

### 4.2 Measures

To ensure the reliability and validity of the measurement tools, we utilized well-established scales widely recognized in academic research, that is mentor support scale developed by Overall et al. (2011), research self-efficacy scale developed by Forester et al. (2004), learning engagement scale developed by Doğan (2014) and research creativity scale by Scott and Bruce (1994). These scales have been widely utilized in educational and psychological research involving graduate students. In China, many scholars have adopted these instruments in their studies, and they have undergone rigorous testing to ensure strong internal consistency, construct validity, and contextual appropriateness (Lin et al., 2019; Ma et al., 2019; Xu and Qiu, 2025; Zhou and Wang, 2024; Yao and Yu, 2019). The selection of these instruments in the present study not only ensures comparability with previous literature but also strengthens the theoretical and methodological foundation of the research. All scales underwent translation and back-translation procedures to ensure linguistic accuracy and cultural appropriateness, followed by expert consultations for optimization. Based on relevant literature, this study sorted out the main variables of mentor support, research creativity, research self-efficacy and learning engagement, and created corresponding questionnaires. After the questionnaires were completed, they were distributed to classmates, mentors, and relevant experts to verify the appropriateness of the measurement items and to check for grammatical errors. Feedback was solicited, and necessary revisions were made accordingly. In line with the educational context of Chinese Ed.D students, this study tailored the questionnaire to their specific learning situations. Finally, the reliability and validity of the instrument were rigorously tested to ensure the robustness of the survey results.

The results of the dimensional reliability tests for the four variables are summarized in Table 2. The overall Cronbach’s alpha coefficient for the Mentor Support scale is 0.880, indicating excellent reliability. Similarly, the Research Self-Efficacy scale shows an excellent reliability with a Cronbach’s alpha of 0.885. The Learning Engagement scale also demonstrates good reliability, with a Cronbach’s alpha coefficient of 0.879. The Research Creativity scale, with a Cronbach’s alpha of 0.870, indicates good reliability as well. Overall, the reliability tests confirm that all scales exhibit acceptable levels of internal consistency.

**Table 2:** Reliability analysis of each scale.

Variables	Dimension	Items	Cronbach’s coefficient	
Mentor support	Learning support	4	0.871	0.880
	Emotional support	3	0.844	
	Academic support	3	0.858	
Research self-efficacy	Curriculum learning	3	0.820	0.885
	Research activity	4	0.875	
	Teaching practice	3	0.849	
Learning engagement	Behavioral engagement	4	0.857	0.879
	Cognitive engagement	3	0.868	
	Emotional engagement	3	0.813	
Research creativity	Research achievements	6	0.870	0.870

In terms of validity analysis, this study examines the Kaiser-Meyer-Olkin (KMO) value. As shown in Table 3, the KMO value is 0.897, and the significance level is less than 0.05, indicating that the data are appropriate for factor analysis. This suggests that the sample size is sufficient and the data structure is suitable for extracting meaningful factors.

**Table 3:** KMO and Bartlett’s test.

**KMO** Measure of Sampling Adequacy		0.897
Bartlett’s test of sphericity	Approx. chi-square	7405.809
	df	630
	Sig.	0

### 4.3 Analysis of model fit

**Figure 2:**
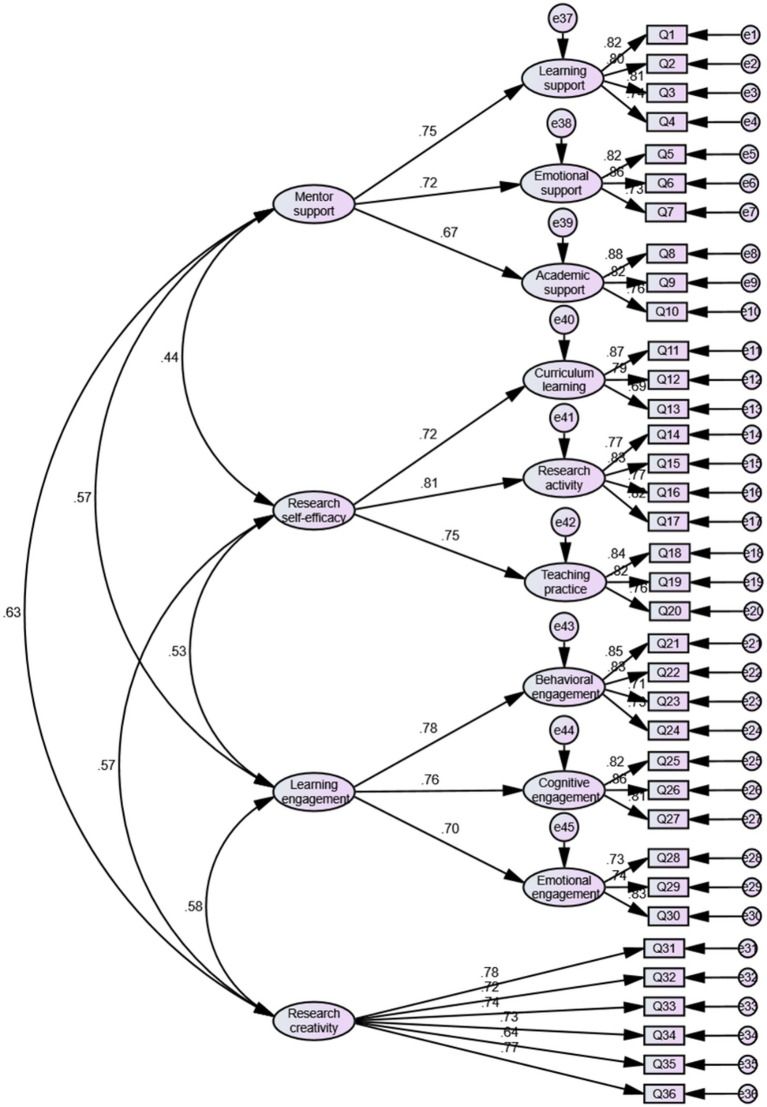
Confirmatory factor analysis results of the model.

To further examine the accuracy of the model in measuring the constructs, a confirmatory factor analysis (CFA) was performed using AMOS 24.0, as illustrated in Figure 2. The analysis yielded strong model fit indices: X^2^/df = 1.488 (<3), RMSEA = 0.037 (<0.08), SRMR = 0.049 (<0.05), GFI = 0.885 (>0.80), CFI = 0.960 (>0.90), TLI = 0.956 (>0.90), and IFI = 0.960 (>0.90) (Hair et al., 2010; Kline, 2016). These values confirm that the model demonstrates a good fit to the data. Furthermore, all standardized factor loadings surpassed the recommended threshold of 0.50 (Hair et al., 2010), reinforcing the validity of the measurement model. The results indicate that the model meets the established criteria, confirming its robustness and structural validity.

### 4.4 Statistical analysis

Data were processed and analyzed using SPSS 26.0 and AMOS 24.0 software. The analytical procedure consisted of several steps: (1) Preliminary Screening: The collected data were initially screened for validity. Questionnaires with unusually short response times or those providing incorrect answers to reverse-coded items were excluded. (2) Descriptive Statistics and Correlation Analysis: Descriptive statistics were computed to summarize the sample characteristics, followed by Pearson correlation analysis to examine the relationships among the study variables. A total of 366 valid questionnaires were collected to test the proposed hypotheses. The scales measuring mentor support, research creativity, research self-efficacy, and learning engagement were analyzed using SPSS 26.0. (3) Structural equation modeling (SEM) was conducted to assess both the measurement and structural models. Confirmatory factor analysis (CFA) was performed to evaluate reliability and validity using factor loadings, composite reliability (CR), and average variance extracted (AVE). Model fit was assessed through goodness-of-fit indices, while path coefficients were examined to validate the structural model. Lastly, the bootstrapping method was employed to test the statistical significance of the proposed mediating effects.

## 5 Results

### 5.1 Difference analysis

This study examined the relationship between demographic variables and key factors, with a particular focus on gender differences, using independent sample *t*-tests. As presented in Table 4, the significance level for mentor support is 0.171, exceeding the 0.05 threshold, indicating no significant gender difference in this variable. However, research self-efficacy has a significance level of 0.004, which is below 0.05, suggesting a significant gender difference, with males reporting higher research self-efficacy on average. Similarly, the significance level for learning engagement is 0.173, indicating no significant gender difference in this dimension. In contrast, research creativity exhibits a significance level below 0.05, confirming a significant gender difference, with males demonstrating higher research creativity.

**Table 4:** Independent sample *T*-test for gender.

Variables	Gender	Number	Mean	Std. deviation	*T*-value	Significance
Mentor support	Male	170	3.67	0.69	1.372	0.171
	Female	196	3.57	0.73		
Research self-efficacy	Male	170	3.62	0.70	2.924	0.004**
	Female	196	3.39	0.77		
Learning engagement	Male	170	3.55	0.72	1.366	0.173
	Female	196	3.45	0.74		
Research creativity	Male	170	3.62	0.86	3.923	0.000**
	Female	196	3.26	0.88		

** Was significantly associated at 0.01 level.

To examine variations in key variables across different grade levels, paired t-tests were conducted for pairwise comparisons. As presented in Table 5, significant differences were observed in mentor support and research creativity across grade levels, following the trend: fourth-year > third-year > second-year > first-year. A similar pattern emerged for research self-efficacy and learning engagement, ranked as fourth-year > first-year > third-year > second-year. Fourth- and first-year Ed.D students reported relatively higher levels of learning engagement, particularly when working on dissertations and coursework. These findings align with traditional academic trajectories, where first-year students primarily focus on foundational coursework, while third- and fourth-year students are more actively engaged in research-oriented activities, such as publishing papers and completing graduation projects. As students progress, increased interactions with mentors provide critical guidance and support, leading to greater academic involvement. Moreover, producing research outputs requires sustained effort and advanced research skills, placing greater demands on students’ competencies. First-year students, with limited research experience, tend to exhibit lower research creativity. However, as students advance, their self-efficacy and research competencies improve, driven by increased involvement in research activities and mentoring support.

**Table 5:** Analysis of variance of grade.

	**Grade (Mean±Std. Deviation)**				*F*	*p*	Post test
	First year (*n* = 89)	Second year (*n* = 94)	Third year (*n* = 92)	Fourth year (*n* = 91)			
Mentor support	3.41 ± 0.74	3.51 ± 0.74	3.71 ± 0.64	3.83 ± 0.64	6.844	0.000**	Fourth year>Third year>Second Year>First Year
Research self-efficacy	3.63 ± 0.73	3.33 ± 0.80	3.34 ± 0.74	3.68 ± 0.63	5.866	0.001**	Fourth year >First year>Third year> Second year
Learning engagement	3.56 ± 0.72	3.32 ± 0.79	3.44 ± 0.69	3.67 ± 0.67	4.083	0.007**	Fourth year>First year>Third year> Second year
Research creativity	3.20 ± 0.95	3.24 ± 0.83	3.50 ± 0.86	3.77 ± 0.81	8.436	0.000**	Fourth year>Third year>Second Year>First Year

** Was significantly associated at 0.01 level.

### 5.2 Descriptive statistics and correlation analysis

The descriptive statistics and correlation analysis results for the study variables are presented in Table 6. The findings indicate that students generally perform well across the four dimensions. The relatively small standard deviation suggests minimal individual differences, indicating that the data are fairly consistent across participants. However, the average score for research self-efficacy is comparatively low, which may be due to the fact that Ed.D students typically publish fewer research papers, resulting in fewer opportunities for successful academic experiences. Similarly, the average score for learning engagement is also relatively low, possibly reflecting limited engagement in research and academic activities among Ed.D students.

**Table 6:** Descriptive statistical analysis and correlation analysis results among the variables.

Variables	Mean value	Standard deviation	Mentor support	Research self-efficacy	Learning engagement	Research creativity
Mentor support	3.615	0.711	1			
Research self-efficacy	3.497	0.745	0.320**	1		
Learning engagement	3.495	0.729	0.413**	0.396**	1	
Research creativity	3.428	0.889	0.489**	0.469**	0.446**	1

**p < 0.01.

The table also indicates that mentor support is positively correlated with research self-efficacy, learning engagement, and research creativity, underscoring its crucial role in fostering these factors. Additionally, research self-efficacy exhibits a positive correlation with both learning engagement and research creativity, suggesting that students with greater confidence in their research abilities are more likely to engage actively in academic activities, leading to enhanced creativity. Furthermore, the significant correlation between learning engagement and research creativity highlights the importance of sustained academic involvement in driving innovation. Overall, mentor support, self-efficacy, learning engagement, and research creativity interact dynamically, collectively shaping students’ academic performance and research outcomes.

### 5.3 Structural model

The study adopted goodness-of-fit indices and path coefficients to evaluate the structural model using AMOS 24.0. According to existing research, a structural model is considered a good fit when X^2^/df falls between 0 and 3, IFI, CFI, TLI, GFI, and AGFI exceed 0.80, and SRMR and RMSEA are below 0.08. As shown in Table 7, the model fit indices were as follows: X^2^/df = 1.488, IFI = 0.960, CFI = 0.960, TLI = 0.956, GFI = 0.885, AGFI = 0.868, SRMR = 0.049, and RMSEA = 0.037. These values confirm that the proposed structural model demonstrates a strong fit to the data, validating its reliability and robustness.

**Table 7:** Goodness of fit index of the structural model.

Index	*x*^2^/df	GFI	AGFI	NFI	IFI	TLI	CFI	RMSEA	SRMR
Suggested value	<3	>0.8	>0.8	>0.8	>0.9	>0.9	>0.9	<0.08	<0.05
Value of this study	1.488	0.885	0.868	0.888	0.960	0.956	0.960	0.037	0.049

**Figure 3:**
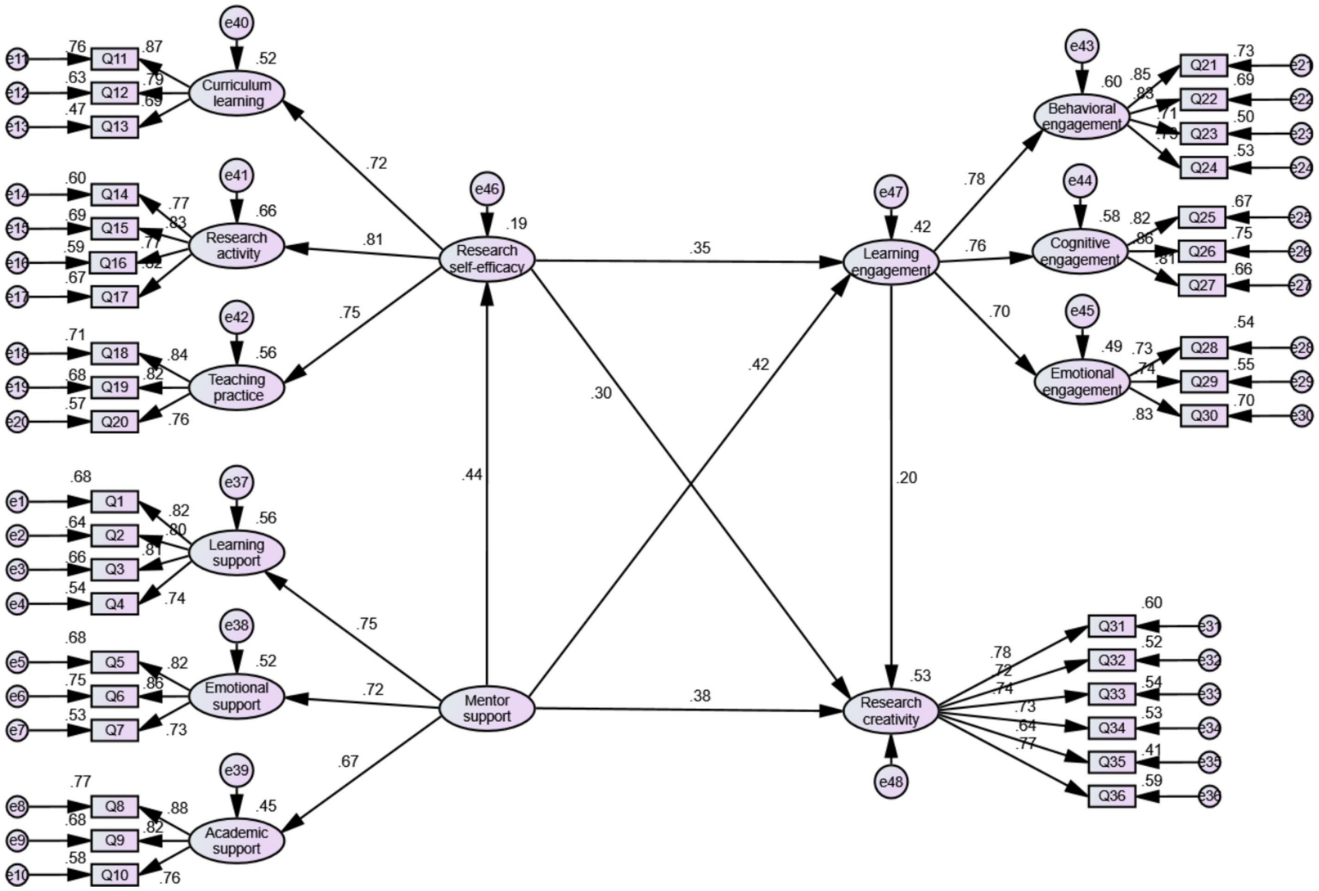
The structural modeling diagram.

Most values met the recommended thresholds, confirming that the alternative structural model demonstrated an acceptable fit. Figure 3 presents the explanatory variance and path coefficients of the model, based on standardized parameter estimates. Mentor support accounted for 19% of the variance in research self-efficacy, with a standardized regression coefficient of 0.440. Additionally, mentor support and research self-efficacy together explained 42% of the variance in learning engagement, with standardized regression coefficients of 0.417 and 0.348, respectively. Furthermore, mentor support, research self-efficacy, and learning engagement collectively explained 53% of the variance in research creativity, with corresponding standardized regression coefficients of 0.384, 0.300, and 0.199, respectively. A bootstrap test with 5,000 resampling confirmed that all path coefficients were statistically significant (*p* < 0.001), providing strong empirical support for the model. These findings validate the alternative structural model, demonstrating its robustness in explaining the relationships among the variables.

### 5.4 Hypothesis tested

Building on the existing literature, several hypotheses were formulated to address the research questions. The hypotheses were tested using AMOS 24.0, with bootstrap sampling employed to examine the mediating roles of various constructs, following contemporary methodological standards (Hussain et al., 2021). The model included direct pathways from mentor support to research self-efficacy, learning engagement, and research creativity. Additionally, indirect effects were assessed using the bootstrap method to estimate confidence intervals, ensuring the robustness of the mediation analysis. The results of these analyses are summarized in Tables 8, 9.

**Table 8:** Structural relationships and hypothesis testing.

**Hypothesis**	**Path**	**Path coefficient**	**t statistics**	***p* values**	**Decision**
H1	Mentor support→research creativity	0.092	4.757	<0.01	Supported
H2	Mentor support→research self-efficacy	0.096	5.411	<0.01	Supported
H3	Research self-efficacy→research creativity	0.069	4.187	<0.01	Supported
H5	Mentor support→learning engagement	0.111	4.971	<0.01	Supported
H6	Learning engagement→research creativity	0.070	2.478	<0.01	Supported
H8	Research self-efficacy→learning engagement	0.089	4.367	<0.01	Supported

**Table 9:** The mediation effects.

**Mediating relationship**	**Estimate**	**SE**	**95% CI lower limit**	**95% CI upper limit**	***P-*value**
Mentor support→research creativity (Direct effect)	0.439	0.100	0.252	0.647	0.000
Mentor support→research self-efficacy→research creativity (H4)	0.151	0.045	0.080	0.259	0.000
Mentor support→ learning engagement→research creativity (H7)	0.095	0.049	0.021	0.218	0.014
Mentor support→research self-efficacy→learning engagement→research creativity (H9)	0.035	0.019	0.008	0.086	0.011
Mentor support→research creativity (Total effect)	0.719	0.098	0.547	0.935	0.000

H1 confirmed a significant positive relationship between mentor support and Ed. D research creativity (*β* = 0.092, *t* = 4.757, *p* < 0.01). Similarly, H2 demonstrated a significant positive association between mentor support and Ed.D research self-efficacy (*β* = 0.096, *t* = 5.411, *p* < 0.01). Furthermore, H3 indicated that research self-efficacy positively influences Ed.D research creativity (*β* = 0.069, *t* = 4.187, *p* < 0.01). H5 established that mentor support is positively linked to Ed.D learning engagement (*β* = 0.111, *t* = 4.971, *p* < 0.01), while H6 confirmed a positive relationship between learning engagement and Ed.D research creativity (*β* = 0.070, *t* = 2.478, *p* < 0.01). Lastly, H8 revealed a significant association between Ed.D research self-efficacy and learning engagement (*β* = 0.089, *t* = 4.367, *p* < 0.01). These findings provide robust empirical support for all six hypotheses, contributing to the study’s research objectives. For details on the mediation effects and outcomes of the mediation analysis, refer to Table 9.

To test the mediation hypotheses, AMOS 24.0 software was used, and the bootstrap sampling method was applied to examine the mediating effects. The key criterion for mediation is whether the confidence interval (CI) includes zero—if it does, the mediation effect is not significant; if it does not, the mediation effect is confirmed. In this study, 5,000 bootstrap resamples were generated with a 95% confidence interval to ensure robust statistical inference. According to the results, in the path “Mentor Support→Research Self-Efficacy→Research Creativity,” the mediating effect of research self-efficacy was 0.151. Since the 95% CI does not include zero, this indicates a significant mediating effect, supporting H4. Similarly, in the path “Mentor Support →Learning Engagement→Research Creativity,” the mediating effect of learning engagement was 0.095, and since the 95% CI does not contain zero, the effect is significant, confirming H7. Additionally, in the chain mediation path “Mentor Support→Research Self-Efficacy→Learning Engagement→Research Creativity,” the indirect effect was 0.035. Given that the 95% CI does not include zero, this demonstrates a significant chain mediating effect, providing empirical support for H9. These findings highlight the critical role of research self-efficacy and learning engagement in mediating the relationship between mentor support and research creativity.

## 6 Discussion

### 6.1 There are differences in the variables among different groups of Ed.D students

This study found that male Ed.D students exhibited higher levels of research self-efficacy and research creativity than their female counterparts. Males demonstrated greater future problem-solving skills (Elballah et al., 2024), which may be attributed to their higher confidence in overcoming research challenges. Men also reported higher self-efficacy in the social sciences compared to women (Huang, 2013). Men are generally expected to display greater competitiveness and autonomy in both academic and professional settings. According to a national survey in China, female researchers lag behind males in both the proportion and quantity of innovative outputs, including published papers, patents, and the transformation of research results (Zhang, 2025). These gendered expectations may influence their level of commitment to education and career development (Wang and Zhang, 2024). Social role theory suggests that boys are often encouraged to develop independence and innovation in their thinking and decision-making, which may lead them to approach academic tasks more holistically and objectively. In contrast, women often experience greater challenges in balancing home and academic responsibilities (Brown and Watson, 2010), particularly those with children, who struggle to allocate sufficient time for research. Women in academia often bear greater emotional and physical responsibilities related to family and childcare, which can increase psychological burdens and further exacerbate their disadvantaged position (Cui et al., 2025; Wu et al., 2025). Female Ed.D students frequently face conflicts between professional and family roles, which restrict the time and energy they can dedicate to academic work, thereby affecting their research productivity. Although the productivity gap between men and women in science has narrowed with the increasing status of women in the field, disparities still exist. These findings contrast with Channing et al. (2023), who reported no significant gender differences in graduation rates or GPAs among doctoral students. This discrepancy underscores the need for further investigation into sociocultural and institutional factors influencing gender disparities in doctoral education.

The analysis of mentor support and research creativity reveals a clear trend: fourth-year > third-year > second-year > first-year. First-year students are primarily focused on adapting to the university environment, taking foundational courses, and having limited exposure to instructor-led research projects. Their understanding of the field is still developing, which results in lower levels of mentor support and research creativity. Second-year students gain some research experience but are still in the process of identifying their areas of specialization and may not interact with their mentors as frequently as their senior counterparts. By the fourth year, students typically work closely with their mentors on thesis research or senior projects, requiring originality, which enhances their research creativity. Regarding research self-efficacy and learning engagement, the ranking follows the pattern: fourth-year > first-year >third-year >second-year. Fourth-year students have accumulated extensive research experience, possess strong confidence in their abilities, and demonstrate high engagement in research. First-year students, driven by curiosity and enthusiasm for their chosen field, also exhibit relatively high engagement (Zhang Y. et al., 2024; Zhang B. et al., 2024). In the first year, students primarily focus on coursework, which allows for greater time investment in learning. The academic pressure is relatively low, making it easier for students to experience a sense of achievement, thereby enhancing their self-efficacy. Third-year Ed.D students often experience significant pressure related to graduation requirements, particularly the completion of their dissertations and uncertainty about academic outcomes. Consequently, their research self-efficacy may decline as a result of increased workload and heightened emotional strain. In contrast, second-year students experience a transitional phase, where they develop research skills but lack the experience and confidence of more advanced students (Zhang Y. et al., 2024; Zhang B. et al., 2024). These variations may stem from the time required to refine academic skills, establish effective study habits, and achieve meaningful academic progress.

### 6.2 Mentor support and research creativity

This study explored the relationship between mentor support and research creativity among Ed.D students, revealing a significant positive correlation. The three dimensions of mentor support—learning support, emotional support, and academic support—were found to positively impact students’ research abilities. These results highlight the pivotal role that mentor support plays in fostering research creativity by providing students with the autonomy to explore research questions and influencing their academic output. This finding is consistent with prior research, which underscores that mentor support enhances students’ research creativity by promoting a positive academic environment (Murphy et al., 2007; Hemmings and Kay, 2016). Additionally, the mentor-student relationship has been identified as a key determinant of research achievement (Smith, 2022).

Social cognitive theory posits that human abilities are shaped by both internal and external factors. In this context, mentor support serves as an essential external factor in developing Ed. D students’ research skills. Constructive feedback and encouragement from mentors are crucial in fostering academic self-efficacy, as individuals with strong mentor support are more likely to believe in their ability to overcome challenges (Bandura, 1978; Liu et al., 2021). This research further emphasizes the importance of mentor support in enhancing academic self-efficacy, which significantly influences students’ overall academic performance. Thus, a supportive mentoring environment is indispensable for nurturing the innovation abilities of Ed. D students by providing them with the necessary resources, guidance, and emotional encouragement.

### 6.3 The mediating effect of research self-efficacy

This study further substantiates the role of research self-efficacy as a mediating factor between mentor support and research creativity among Ed.D students. Consistent with the core principles of social cognitive theory, this research highlights self-efficacy as a pivotal mediator in the relationship between mentor support and research creativity. Social cognitive theory, as articulated by Bandura (1997), underscores the significance of self-belief in determining how individuals approach academic tasks, regulate their behaviors, and overcome challenges. In this context, our findings reveal that Ed.D research self-efficacy partially mediates the relationship between mentor support and research creativity. This outcome aligns with prior studies, which have shown that as students’ research self-efficacy increases, their academic performance improves, due to heightened confidence in their ability to navigate and surmount research challenges (Sawitri and Creed, 2021; Affuso et al., 2023; Cai et al., 2023). Ed.D students who possess a strong sense of research self-efficacy exhibit greater confidence in their ability to generate innovative ideas, formulate critical research questions, and persist in problem-solving endeavors. These behaviors significantly contribute to enhanced research creativity and, consequently, to better research outcomes.

### 6.4 The mediating effect of learning engagement

The results of the study indicate that learning engagement serves as a significant partial mediating variable between mentor support and Ed.D research creativity. This finding aligns with prior research highlighting the critical role of learning engagement in fostering academic creativity, underlining the importance of active involvement in achieving academic success (Mayer et al., 2009; Boelens et al., 2017). Additionally, the present findings are consistent with research suggesting that learning engagement contributes to the creation of a supportive social environment, which can enhance academic motivation and sustained involvement in learning (Bakker et al., 2012; Chen et al., 2024). Students who demonstrate high levels of learning engagement are better equipped to exhibit resilience and perseverance in the face of challenges, leading to more active participation in their academic pursuits. Furthermore, learning engagement has been shown to mediate the relationship between social support and academic performance, highlighting its central role in promoting favorable educational outcomes (Carmona-Halty et al., 2021; Tao et al., 2022). This mediating function extends to the effect of perceived teacher or mentor support on student achievement, emphasizing that mentor guidance can have a direct impact on student engagement, which in turn boosts academic performance.

### 6.5 The chain mediating role of research self-efficacy and learning engagement

The results of this study highlight the chain mediating role of research self-efficacy and learning engagement in the relationship between mentor support and research ability among Ed.D students. Mentor support plays a crucial role in enhancing Ed.D students’ self-efficacy by providing guidance, resources, and emotional encouragement, which collectively boost students’ confidence in their ability to conduct research. This finding aligns with El-Sayad et al. (2021), who emphasized that perceived social support, including mentorship, is fundamental in enhancing self-efficacy, motivation, and academic performance. Once self-efficacy is strengthened, students are more likely to exhibit higher levels of learning engagement, which encompasses the time, effort, and cognitive resources invested in academic tasks (Curtin et al., 2016). This increased engagement correlates with improved academic outcomes, such as greater research creativity and problem-solving capabilities. Engaged students are better equipped to persist in the face of challenges and pursue innovative solutions to academic problems (Zacher and Johnson, 2015). The findings suggest that mentor support positively influences Ed.D students’ research creativity by enhancing both their self-efficacy and learning engagement. This chain mediating effect is consistent with previous studies indicating a strong link between self-efficacy, learning engagement, and academic achievement (Wang et al., 2023). In conclusion, the mediating roles of research self-efficacy and learning engagement illustrate how mentor support fosters greater engagement and creativity in Ed.D students’ research endeavors.

## 7 Conclusions and implications

### 7.1 Conclusion

This study offers a comprehensive understanding of the roles that mentor support and individual factors, such as research self-efficacy and learning engagement, play in shaping the research creativity of Ed.D students. By establishing a chain mediating model, the research bridges the gap between environmental and individual factors, highlighting how mentor support within academic institutions enhances the research creativity of Ed.D students. The findings underscore the importance of nurturing and supporting mentor behavior, as it is a critical factor in fostering the research capabilities of Ed.D students. Mentors play a vital role not only in the academic development but also in the personal growth of their students. Specifically, mentor support significantly boosts both research self-efficacy and learning engagement. As a key social factor, mentor support interacts with student self-efficacy to sustain engagement (Barratt and Duran, 2021; Liu et al., 2018). Mentors contribute by providing academic guidance, sharing experiences, and helping students navigate research challenges, thus enhancing students’ confidence and engagement. Furthermore, the study demonstrates that mentor support positively impacts the research creativity of Ed.D students through the chain mediating effects of research self-efficacy and learning engagement.

### 7.2 Implications

This study contributes to the theoretical discourse on graduate education by empirically validating and extending social cognitive theory within the context of Ed.D programs. The findings reinforce Bandura’s (1997) assertion that self-efficacy is shaped by social and environmental influences. Specifically, the study demonstrates that mentor support significantly enhances Ed.D students’ research self-efficacy and research creativity. Furthermore, by confirming the mediating effect of learning engagement, the study positions engagement not merely as an outcome but as a process variable—a mechanism through which psychological resources are transformed into tangible academic outputs.

This study also offers several practical implications. Firstly, universities should focus on cultivating a supportive research environment by establishing academic exchange platforms and enhancing graduate students’ self-efficacy in research. Environments that provide support and help students derive a sense of personal accomplishment and satisfaction from their work are more likely to promote efficacy (Livinƫi et al., 2021). The Ed.D program is designed to support students who already possess rich professional experience by deepening their theoretical knowledge to better guide and improve their educational practices. Therefore, universities should emphasize both the practical orientation and applied value of the curriculum, ensuring that the content aligns with students’ actual needs and expectations. ChatGPT offers significant applications in higher education by providing continuous, ondemand support, personalized tutoring, enhanced revision tools, and accessibility aid, especially benefiting students who require flexible learning options (Ravšelj et al., 2025). Given the relatively weak academic foundation of many Ed.D students, offering online courses can help strengthen their foundational knowledge and improve their digital competence, thereby enhancing their engagement in learning. This ensures that Ed.D students have sufficient time and energy to devote to their academic pursuits. Given the relatively low scientific research self-efficacy among Ed.D students, universities should design a well-balanced curriculum that includes academic discussions and research training. In addition, female Ed.D students should be encouraged to participate more actively in academic conferences and research cooperation projects to expand the scope and quality of their collaborations, thereby broadening their academic networks.

Secondly, mentor support can manifest in various ways, including providing easy access to information, facilitating interpersonal communication and interactions, offering constructive feedback, and tailoring approaches to individual learning needs. Ed.D students often experience a sense of isolation during their studies and face learning challenges. The frequency and timing of communication between Ed.D candidates and their mentors are often constrained by practical conditions, which can negatively impact students’ progress and delay their graduation (Tan et al., 2022). A mentor’s research experience, frequency of supervision, and the mentor’s preferred supervisory style and methods—all significantly influence a student’s academic achievement (He and Zhu, 2023). Mentors should actively encourage Ed.D students to participate in various academic exchange activities and introduce them to field experts when appropriate, thereby helping them establish broader academic networks. Mentors offer doctoral students extensive opportunities to participate in research and access valuable resources, which significantly accelerates their professional socialization process (Zhou and Wang, 2024). Several studies highlight the potential of ChatGPT usage in higher education, especially for supporting assessment preparation, argumentative writing, research and analysis, programming, and scientific writing (Ravšelj et al., 2025). Mentors can suggest integrating ChatGPT into students’ independent study routines to reinforce learning engagement. In addition, mentors’ psychological support runs throughout the entire doctoral training process and exerts a significant influence on various aspects of doctoral students’ research development. When Ed.D students encounter challenges during research activities that lead to psychological strain, mentors should proactively communicate with them to help alleviate their stress and restore a balanced mental state. This helps students develop a clear understanding of research-related pressure and coping strategies. Such supportive practices can contribute to a more nurturing academic environment that fosters research self-efficacy and engagement among students.

Lastly, students should actively foster learning engagement by participating in academic exchanges, discussions, and collaborative learning activities. Such engagement enables students to deepen their understanding of their field, identify gaps in their knowledge, and enhance their problem-solving skills. Increased learning engagement, particularly in immersive settings, can significantly improve students’ scientific research abilities. However, many Ed.D students balance their studies with professional work, which limits their time for full-time study. Their learning continuity is often disrupted by administrative duties or everyday tasks, leading to reduced engagement and lower academic achievement. To address these challenges, Ed.D students should prioritize communication and exchange with peers and mentors, actively engage in academic activities such as discussions, and continually work to enhance their effective learning engagement. These efforts can ultimately boost their research creativity and academic performance.

## 8 Limitations and future research directions

This study provides valuable insights into how mentor support influences the research creativity of Ed.D students, with research self-efficacy and learning engagement functioning as chain mediators. However, it is not without limitations. First, the small sample size may limit the generalizability of the results. Additionally, the use of a one-dimensional scale to measure research creativity oversimplifies the construct, potentially overlooking its complex and multifaceted nature. Moreover, the study’s methodology does not fully explore the intricate, nonlinear relationships among the various influencing factors. Future research should address these limitations by using larger, more diverse samples, employing advanced analytical techniques, and developing a multidimensional scale to measure innovation ability. As the sample was drawn from two leading “Double First-Class” universities, the findings may not generalize to other contexts. Future research should include diverse institutions to better understand how mentor support, self-efficacy, and engagement shape Ed.D students’ research creativity across varying settings. Moreover, qualitative approaches such as in-depth interviews or focus group discussions could capture the contextual factors that shape the mentor-mentee relationship. Longitudinal studies could provide valuable insights into how mentor support, research self-efficacy, and learning engagement evolve over time and influence long-term research creativity. These methods would offer a more comprehensive and temporally sensitive understanding of the mechanisms underlying Ed.D student development.

## Data Availability

The original contributions presented in the study are included in the article/supplementary material, further inquiries can be directed to the corresponding author/s.
